# *Coriandrum sativum* seeds extract mitigate progression of diabetic nephropathy in experimental rats *via* AGEs inhibition

**DOI:** 10.1371/journal.pone.0213147

**Published:** 2019-03-07

**Authors:** Anu Kajal, Randhir Singh

**Affiliations:** M. M College of Pharmacy, M. M (Deemed to be University), Mullana, Ambala, Haryana, India; Alexandria University, EGYPT

## Abstract

Inthe present study, we have demonstrated the phytochemical composition of petroleum ether extract of *C*. *sativum* (CPE) seeds by using chromatographic, spectroscopic as well spectrometric analysis. CPE was evaluated for its possible role in mitigation of diabetic nephropathy (DN) in Streptozotocin (STZ)-nicotinamide (NAD) induced type 2 diabetes model. Administration of CPE at doses of 100, 200, and 400 mg/kg for 45 days has produced significant attenuation of elevated biochemical parameters including serum glucose, lipid and creatinine levels. CPE has also reserved albuminuria and elevated creatinine clearance in treated diabetic rats. Advanced glycation end products (AGEs) formation in kidneyswas also considerably reduced along with noteworthy increase in level of superoxide dismutase (SOD), glutathione (GSH), and decrease in lipid peroxidation in terms of thiobarbituric acid reactive species (TBARS). Molecular docking studies were also employed to reveal out the possible mechanism. In conclusion, using STZ-NAD model, we have successfully predicted that by assets of bioactive constituents CPE might inhibit the progression of DN. *C*. *sativum* may act as potential adjuvant for antidiabetic therapy and needs to be investigated further.

## Introduction

Hippocrates, the founder of *medicine as arational science* has studied many medicinal properties of foods and summarized *food asmedicine*. Many traditional systems including Ayurveda and traditional Chinese medicine systems are practising use of food as medicine throughout history. For being medicinally active or for being functional, a food should contain biologically active componentswhich contribute to enhanced health or reduced risk of disease apart from being nutritious. These are well thought out as foods which are part of a normal diet[1,2,3].

*Coriandrum sativum* L. is an important member of Apiaceaefamily.It is identified as one of the oldest herbal plants valued for its nutritional and medicinal properties[4].Leaves and seeds of this plant are used as spice as well astherapy for various disorders in traditional medicine systems[5].*C*. *sativum* is a potential source of multiple bioactive componentssuch as flavonoids(quercetin and isoquercetin, rutin), terpenes (linalool), terpenoids, polyphenols (ferrulic acid, gallic acid, rutin, caffeic acid, anethole, borneol, caroteinoids), fatty acids rich in petroselinic acid, coumarins and hydroxy-coumarins, tannins, sterols vitamin C and tocopherols etc. which may be responsible for its therapeutic action [4,6].*C*. *sativum* possesses interesting pharmacological activities such asantidyslipidemic, anti-inflammatory, cardio and neuroprotective etc. [7,8].*C*. *sativum*may be consideredas a functional food due to itscommon used in regular diet. Moreover, its pharmacological benefits in various disorders promote its use as a functional food [7].

Diabetic nephropathy (DN) is one of the leadingcauses of mortality in diabetic patients. DN is generally characterized by the thickening of basement membrane, expansion of messengial cells, fibrosis and apoptosis of podocytes which leads tostructural as well as functional abnormalities. The functional impairment of renal cells leads to increased excretion of urinary albumin, urea, uric acid and creatinine, BUN, fluid retention, glomerular lesions and reduce glomerular filtration rate (GFR) in diabetic patients [9]. Furthermore, chronic hyperglycemia and oxidative stress are two major culprits for onset of complications related to diabetes[10,11]. Hexokinase enzyme phosphorylates glucose for energy production under normal conditions whereas chronic hyperglycemia heads to saturation of hexokinase. This saturation results to entrance of excess glucose to pathways such aspolyol pathwayand ultimately leads to increased formation of AGEsand their receptors [10,11].The interaction between AGEs and their receptors causes vascular aging and damage, consequently involved actively in the pathogenesis of DN. Chronic hyperglycemiaalso induces oxidative stress by increasingthe level of reactive oxygen species (ROS), which facilitates formation of AGEs. Therefore, alterations in above mentioned biochemical pathways lead to structural and functional aberration of nephrons and eventuallyDN.

Along with the available interventions of diabetes like oral hypoglycemic agents and insulin therapy, many diabetic patients also choose complementary and alternative therapies which have intensified the demand for alternative therapies for management of diabetes [12].Following the direction of using safenatural products along with functional foods, their bioactive constituents and medicinal properties, the present study was designed to investigate petroleum ether extract of *C*. *sativum* for attenuation of DN.

## Materials and methods

### Preparation of CPE and phytochemical screening

The plant material of *C*. *sativum* (Brand name: Golden Harvest; Batch no. RAI-1272; Description: Coriander whole) was procured from local market of Ambala (geographical coordinates: Latitude: 30°21′39″ N; Longitude: 76°47′52″ E), Haryana, India and authenticated by Dr. K. Madhava Chetty, Department of Botany, SVU, Tirupati. Plant specimen voucher no. 1078 is available with the institute’s herbariumfor future reference. Seeds of *C*. *sativum*were coarsely powdered and subjected to soxhlet extraction using petroleum ether (60–80°C). The extract was distilled off and concentrated under reduced pressure using rotary evaporator at a temperature of 40°C. This crude extract was used for present study.The phytochemicals present in the CPE were screened using qualitative assays described in Trease and Evans, 1989 [13].

### Total terpenoid estimation

Total terpenoid content in CPE was determined using method described by Ghorai*et al*., 2012 [14]. Linalool was used as a standard. A standard curve was plotted using Linalool (10–500 μg/ml). The concentration of total terpenoid in the sample was obtained as μg of Linalool equivalent per 200 μl of CPE.

### Thin layer chromatography (TLC)-Bioautography

TLC was run using two solvents*i*.*e*., toluene and ethyl acetate in ratio of 7:3 for separating the constituents of CPE. Bioautography was performed to confirm the presence of antioxidant compounds in CPE according to method described by Hemalatha et al., 2016 [15]. The specific compounds which possess antioxidant properties show clear zone of yellow colour against purple background developed by DPPH.

### GC-MS analysis

CPE was derivatised to trimethylsilyl derivatives and subjected to GC-MS analysis.Initially column was maintained at 60 ^o^C for 5 min after injecting the sample and temperature conditions were raised to 140 ^o^C with a proportion of 10 ^o^C per min. Temperature was further increased to 200°C with 5°C per min for 20 minutes. Final temperature of injector was 250°C andfor detector was 275°C. H_2_ was used as carrier gas. Inlet pressure 45 psi linear, gas velocity 39 cm/sec, column flow rate 2.4 mL/min, split ratio (40:1) andinjector volume 1μL.

### High performance liquid chromatographic (HPLC)analysis

HPLC analysis was carried out by using CyberLab (Millibury, USA) HPLC system equipped with 5 μmCapcell Pak C18 column (4.6 mm (ID) × 250 mm, 5 μm) and UV detector. The solvent A was acetonitrile and the solvent B was water in ratio of 55:45, v/v. The gradient elution at 25°C was performed, with flow rate 1.0 ml per minute whereas the detection was done from a range of 190 nm to 280 nm, varying according to the retention time of component.

### UV-Visible spectroscopy

CPE was evaluated for UV-Visible spectral property at a concentration of 0.1 mg/ml by using 2375- Double beam UV-VIS spectrophotometer (Electronics India) for the wavelength range 190–800 nm.

### Fourier transform infra-red (FTIR) spectroscopy

The pellets of CPE and KBr (potassium) were made in a hydraulic press and were subjected to FTIR analysis in range of 450–4000 cm^-1^ using FTIR-Shimadzu spectrometer.

### Molecular docking

Molecular docking was performed using VLife Molecular docking software version 4.6.10. RAGEs (PDB ID: 3CJJ) crystal structure was used for molecular docking. PDB was obtained from RCSB PDB-101 protein crystals database (PDB,http://www.rcsb.org/pdb/). While inserting the PDB in working surface of VLife MDS, all the water molecules were removed. Ligand was prepared in ChemSketch 2017 and was saved as mol.2 file. Protein was analysed for the active site and docked with ligand to generate the docking score. The docked complex was viewed in “interactions” section of the software.

### Animal experiments

Animal experimental protocol was approved bythe Institutional Animal’s Ethics Committee (IAEC) (1355/PO/Re/L/10/CPCSEA) of M. M. College of Pharmacy and was in accordance with guidelines of regulatory body of the government (MMCP/IAEC/16/07). Male Wistar rats weighing 250–300 g were procured from NIPER, Mohali and acclimated at local animal house conditions with temperature 24 ± 2 ^o^C, humidity 45 ± 5%, and 12 h light and dark cycle. All animals had free access to diet and water.

Freshly prepared STZ (by single *i*.*p*. injection of 65 mg/kg) was administered after 15 min of NAD injection (230 mg/kg, *i*.*p*.) for induction of type 2 diabetes(TIIDM). The diabetogenic action of STZ is related to its selective destruction of pancreatic β-cells by oxidative stress *via* increased production of ROS [16]. NAD is an active water soluble form of vitamin B_3_and possessesantioxidant functions. Animals were injected with NAD for its partial protective effect on pancreatic β-cells against STZ, therefore leads to TIIDM. STZ inhibits antioxidant enzymes along with increased levels of lipid peroxidation, which plays a key role in pathogenesis of DN[17]. Therefore, STZ-NAD induced TIIDM model was selected for the study.

Blood glucose level was estimated after 72 h of STZ administration. Animals were fasted overnight for blood glucose estimation and animals with glucose levels more than 250 mg/dL were selected for the study. In case of animals, the major symptom of DN develops over a period of one month i.e. 30 to 45 days. After 30 days of STZ administration, levels of serum creatinine, urea, uric acid, BUN as well as albumin in urine and creatinine clearance were significantly high suggesting development of DN.

#### Selection of doses for CPE and grouping of animals

Different doses for CPE (100, 200 and 400 mg/kg) were selected on the basis of oral acute toxicity studies performed by Patel et al., 2012[18] and also followed the criteria not to test extracts at doses that are not liable to have practical utility[19]. Total numbers of animals taken were eight per group. However, the data was calculated for six animals each group due to immortality. Animals were divided into six groups. Group 1 for normal control; Group 2 for DN control; Group 3 for DN rats receiving 100 mg/kg dose of CPE; Group 4 for DN rats receiving 200 mg/kg dose of CPE; Group 5 for DN rats receiving 400 mg/kg dose of CPE and Group 6 for DN rats receiving 10 mg/kg dose of Glimepiride.

Doses were prepared in suitable vehicle and were administered with the help of oral gavageonce daily and the treatment with extract and glimepiride was continued for 45 days after confirmation of DN onset.

#### Serum and urine biochemical assays

Biochemical estimation of serum glucose, insulin, liver enzymes, lipid profile and renal functionwas carried out using estimation kits of Reckon Diagnostics Pvt. Ltd.

Urine was collected from metabolic cages for a period of 12 h. Urinary creatinine and albumin excretion (UAE) were assessed using kits by Reckon Diagnostics Pvt. Ltd. INDIA. Creatinine clearance (Ccr) was calculated using formula:

Ccr(ml/min/100gbodyweight)=[urinecreatinine(mg/dL)×urinevolume(ml)/serumcreatinine(mg/dL)]×[100/bodyweight(g)]×[1/720(min)].

#### Estimation of antioxidant enzyme levels, AGEs in kidney and histopathological studies

Animals were sacrificed by cervical dislocation at the end of the study and kidneys were obtained. Using rotary microtomes, small sections measuring 5μm thickness were prepared and stained with hematoxylin and eosin (H & E) dye for microscopic observations. Kidney homogenate was used to estimate antioxidant enzymes *viz*. SOD (U/mg protein) and GSH (μM/mg protein). Lipid peroxidation was measured as TBARS(nmol/mg protein).

The levels of AGEs were also estimated in kidneys [20].Briefly, perfused kidneys were homogenized in 2 ml of 0.25 M sucrose followed by centrifugation at 900 g at 5˚C and the supernatant was separated. The pellet was resuspended in 2 ml sucrose solution and centrifuged. The supernatant obtained was mixed with the previous one. The proteins present were precipitated by adding equal volume of trichloroacetic acid (TCA). After centrifugation at 4˚C with 900 g, the protein pellet obtained was mixed with 1 ml methanol twice to remove the lipid fraction. The insoluble protein, after washing with 10% cooled TCA was centrifuged and the residue was solubilized in 1 ml of 1 N NaOH and the protein concentration was estimated by measuring the absorbance at 280 nm against BSA standard curve. The AGEs content was then measured fluorometrically with an emission at 440 nm and excitation at 370 nm, and the results were expressed as relative fluorescence units (RFU)/mg protein.

### Statistical analysis

Statistical analysis was performed using Graph pad Prism 6. Values were expressed as mean ± SEM and one-way analysis of variance (ANOVA) was used for statistical analysis. ANOVA was followed by Tukey’s as *post hoc* multiple comparison test. The results were considered significant if *p* ≤ 0.05. Alphabets (a,b,c,d) were assigned for comparison of different groups. Normal control vs DN control (a); DN control vs 100, 200, 400 mg/kg of CPE and standard drug (b); 100 mg/kg vs 200 and 400 mg/kg (c); 200 mg/kg vs 400 mg/kg (d); *p< 0.001, #*p*< 0.01, †*p*< 0.05

## Results and discussion

### Phytochemical composition

Preliminary phytochemical screeningrevealed the presence of terpenoids, fats and fixed oil. Results so obtained are in accordance with available literature evidencing the presence of terpenoids and fatty acid content in CPE[4].Total terpenoid content of CPE was calculated by plotting standard curve of Linalool; a monoterpene. Total terpenoid content was found to be 37 μg of Linalool equivalent to 200 μl of CPE.

CPE with a concentration of 1mg/ml was run on TLC using toluene and ethyl acetate. Developed TLC plate was dried and visualized under UV light. Two spots at R_f_ value of 0.41 and 0.62 respectively were observed (**S1 Fig**). Furthermore, the developed and dried TLC plate wassprayed with 0.2% DPPH. Reduction of DPPH radical to non-radical DPPHH was confirmed by visual change in colour from purple to yellow indicating the presence of antioxidant compounds. Yellow spots against a purple background were detected on the TLC-bioautograph revealing the richness of antioxidant compounds in CPE (**S2 Fig)**.

Taking into account the results of phytochemical screening, CPE was further subjected to GC-MS analysis for confirmation of activephytoconstituents.The result (peaks) obtained from GC-MS analysiswas interpreted according to data base of National Institute Standard and Technology (NIST-14) library. The identified compounds along with percent composition in CPE are listed in **Table 1**. Results revealed out the presence of Linalool, a monoterpene, ascorbyl palmitate (fat soluble form of ascorbic acid) and different fatty acids in CPE.

**Table 1:** GC-MS analysis result of *C*. *sativum* petroleum ether extract (CPE).

Peak	Retention time	Area %	Database/NIST 14 library
1	7.87	2.39	Linalool
2	18.64	11.53	Ascorbyl Palmitate
3	20.40	69.38	Petroselinic acid
4	20.53	2.22	Docosahexanoic acid
5	22.08	2.56	Linoleic acid
6	23.62	1.62	α-Linolenic acid
7	24.95	10.30	Oleic acid

Linalool was quantified by HPLC analysis and calculated concentration of linalool was expressed in mg/g of CPE. The chromatogram obtained for CPE was identified with a separate distinct peak for linalool at RT 6.723 min when compared with RT of standard linalool at 6.784 min (**S3 Fig**). The concentration of linalool was found to be 43.62 mg/g of CPE.

CPE was also subjected to UV-Visible spectroscopy and the chromatogram obtained has shown 4 major peaks with maximum absorbance near 224, 335, 406 and 674 nm. The absorption patterns were compared with pre-existing data and reports of other investigators for validation and interpretations of terpenes [21]and fatty acids [22] and found to be near similar evidencing the presence of fatty acids and terpenes in CPE.

Furthermore, CPE was characterized by FTIR to identify functional groups distribution. Different predominant functional groups namely hydroxyl, alkane and alkenes were detected by analyzing the FTIR spectrum. The peak values for CPE in FTIR spectrum were obtained at 455.20, 594.08, 725.23, 802.39, 933.55, 1002.98, 1172.72, 1234.44, 1365.6, 1458.18, 1612.49, 2353.16, 2723.49, 2870.08, 2947.23, 3379.29 cm^-1^.

### Improvement inbody weight

**Fig 1:**
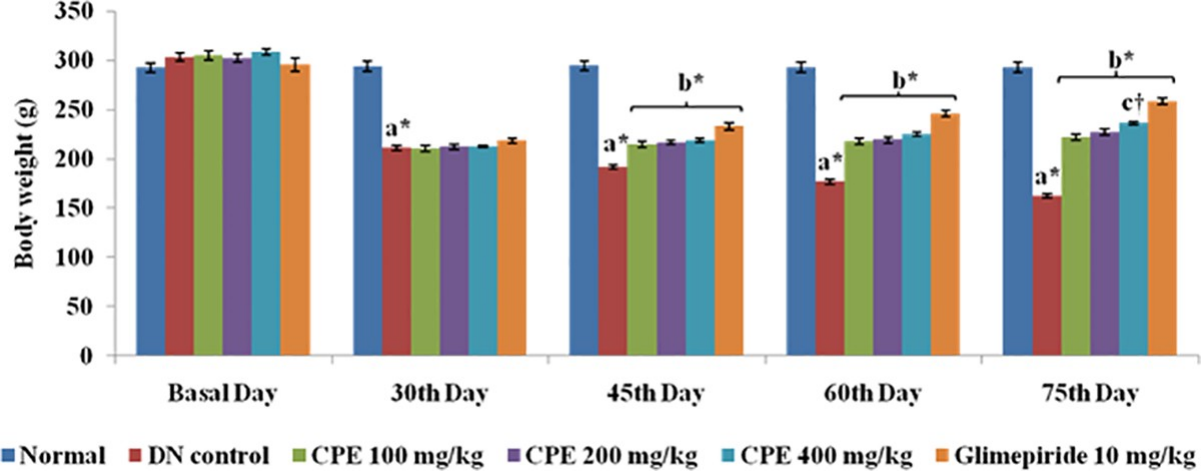
Effect of CPE on body weight (g). Values are represented as Mean ± SEM (n = 6).

STZ induced diabetes is associated to loss of body weight due to increased muscle wasting and catabolism of proteins as a result of chronic hyperglycemia [23].There was no considerable difference observed in body weight of normal control group (284.76 ± 4.51 to 290.17 ± 5.20 g) at the end of study. However, DN control rats were observed with a significant loss in body weight (303.00 ± 4.11 g to 172.87 ± 2.38 g).Moreover, administration of CPE (100, 200 and 400 mg/kg) and glimepiride has improved weight loss in comparison to DN rats in a dose dependant manner. This improvement may be ascribed to inhibition of glucose output from the liver as well as hepatic gluconeogenesis which is also accompanied with the suppression of lipolysis in adipose tissue. Glimepiride also inhibited weight loss in rats (**Fig 1**). Chronic hyperglycemia leads to increased water and food intake in DN control animals as compared to normal control which was significantly improved on oral administration of different doses of CPE.

### Reduction ofserum glucose level

**Fig 2:**
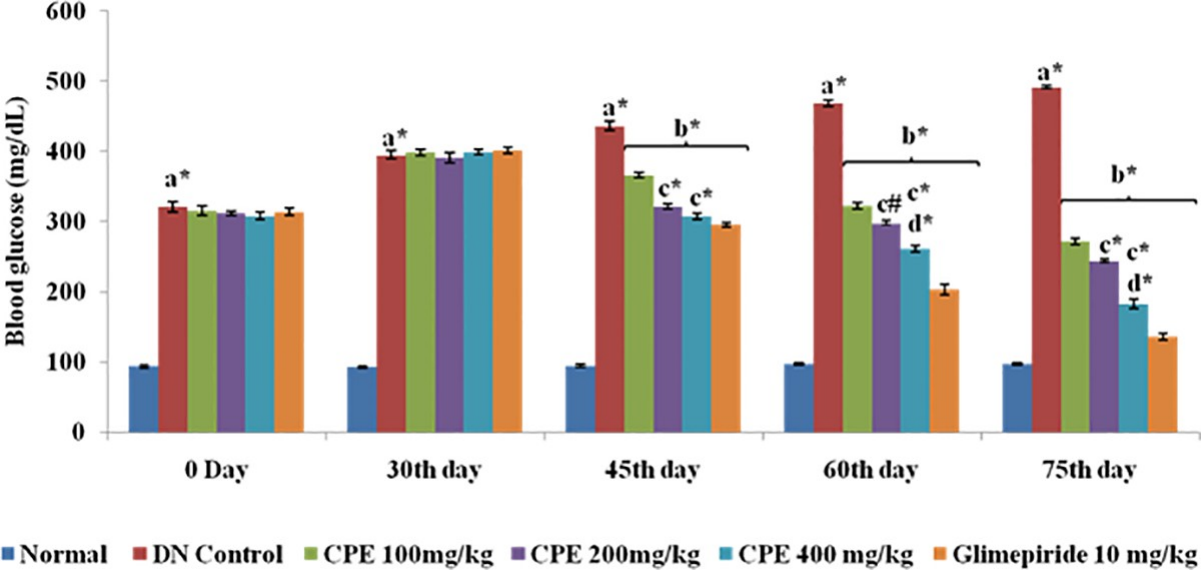
Effect of CPE on blood glucose level (mg/dL). Values are represented as Mean ± SEM (n = 6).

Serum glucose level was significantly increased in DN rats in comparison to normal rats. Oral administration of CPE (100, 200 and 400 mg/kg) produced significant reduction in serum glucose level to 271.00 ± 4.83, 244.01 ± 3.12 and 182.73 ± 4.28 mg/dL respectively in comparison to DN control rats. Effects produced by CPE were found to be dose and time dependant**(Fig 2).** Glimepiride treated group also resulted in significant attenuation of fasting blood glucose level (135.41 ± 4.39 mg/dL) as compared to DN control group (420.23 ± 5.46 mg/dL). The attenuating effects of CPE on glucose level may be attributed to presence of active constituents such as linaloolwhich is traditionally known for its antidiabetic activity.

### Improvement in serum insulin level

The phytoconstituents like terpenes and terpenoids are well known for reducing oxidative stress, hyperglycemia, hyperlipidemia and for secreting insulin from pancreatic β-cells as well as regeneration of β-cells[10]. Serum insulin level was measured at start of study i.e. 30^th^ day and at the end of study i.e. 75^th^ day. On 30^th^ day, serum insulin level was found to be decreased i.e. 6.93 ± 0.26 μIU/mlin DN control rats in comparison to normal control rats i.e. 15.06 ± 0.41μIU/ml. Oral administration of 100, 200 and 400 mg/kg doses of CPE and glimepiride for 45 days has produced significant improvementin serum insulin level to 7.20 ± 0.15, 8.03 ± 0.31, 10.38 ± 0.23 and 11.87 ± 0.27μIU/ml respectively. Our results were supported by studies ofGray and Flatt, 1999 and Eidi et al., 2009 [24,25] foranti-hyperglycemicpotential of *C*. *sativum* by stimulating release of insulin from the pancreatic β-cells.

### Improvement in serum lipid profile

DN is not limited to altered metabolism of glucose but also associated with dyslipidemia, especially in TIIDM[26]. Elevated serum total cholesterol (TC), triglyceride (TG), low density lipoprotein (LDL) and reduced high density lipoprotein (HDL) levels were observed after administration of STZ in diabetic groupswhen compared to normal rats (**Table 2**). These lipid abnormalities contribute to DN*via* activation of inflammatory mediators which are responsible for extracellular matrix protein accumulation in renal cells and ultimately lead to cell damage [10].Coriander seeds are a potential source of mono as well as polyunsaturated fatty acids which was confirmed by GC-MS analysis. Petroselinic acid in CPE possesses anti-inflammatory activity by inhibiting Cox-1 and Cox-2 enzymes [5]. Omega-3 [Docosahexanoic acid (DHA) and alpha-linolenic (ALA)] and omega-6 fatty acids (linoleic acid) promote secretion of anti-inflammatory agents as well as known to reduce TG levels, increase HDL level, improvement in endothelial dysfunction etc.[27].Oral administration of different doses of CPE has significantly reduced the altered level of lipids and thus demonstrates role of CPE in improving lipid metabolism. Similarly, glimepiride has also shown significant effects on lipid profile.

**Table 2:** Effect of *C*. *sativum* petroleum ether extract (CPE) on biochemical parameters in diabetic nephropathy rats.

Groups	Normal control	DN control	CPE 100 mg/kg	CPE 200 mg/kg	CPE 400 mg/kg	Glimepiride 10 mg/kg
TC (mg/dL)	102.33 ± 2.04	299.55 ± 2.10 ^a^*	215.12 ± 0.82 ^b^*	179.03 ± 1.42 ^b^*^c^*	159.65 ± 2.02^b^*^c^*^d^*	151.83 ± 2.19^b^*
LDL (mg/dL)	36.50 ± 1.64	233.38 ± 2.21 ^a^*	133.25 ± 1.27^b^*	110.87 ± 1.19^b^*^c#^	90.43 ± 1.78^b^*^c^*^d^*	94.90 ± 1.59^b^*
TG (mg/dL)	73.67 ± 1.41	205.80 ± 1.61^a^*	128.78 ± 1.35^b^*	117.49 ± 0.82^b^*^c#^	106.24 ± 0.89^b^*^c^*^d#^	100.13 ± 1.92^b^*
HDL (mg/dL)	57.50 ± 0.96	27.44 ± 0.77^a^*	36.45 ± 0.76 ^b^*	40.36 ± 0.29^b^*^c†^	45.71 ± 0.36^b^*^c^*^d#^	36.17 ± 0.80^b^*
AST (IU/L)	37.01± 1.21	84.75± 2.58 ^a^*	70.15± 1.33 ^b^*	58.33± 2.50 ^b^*^c#^	46.23± 1.80 ^b^*d*	45.39± 1.09 ^b^*
ALT (IU/L)	29.41± 0.71	72.02± 2.08	66.28± 1.33 ^b^*	56.38± 2.53 ^b^*^c#^	39.09± 1.81 ^b^*^d^*	36.98±1.43 ^b^*
Urea (mg/dL)	34.73 ± 0.95	89.33 ± 1.41 ^a^*	58.39 ± 0.78^b^*	52.13 ± 1.30^b^*^c^*	48.52 ± 1.18^b^*^c^*^d†^	55.50 ± 1.26^b^*
Uric acid (mg/dL)	5.07 ± 0.13	15.10 ± 0.38 ^a^*	8.39 ± 0.12^b^*	7.97 ± 0.16^b^*	7.36 ± 0.25^b^*^c#d†^	7.67 ± 0.18^b^*
Creatinine (mg/dL)	0.70 ± 0.11	3.92 ± 0.03 ^a^*	2.96 ±0.07^b^*	2.56 ± 0.03^b^*^c†^	2.16 ± 0.05^b^*^c^*^d^*	1.56 ± 0.07^b^*
BUN (mg/dL)	16.73 ± 0.44	41.72 ± 0.66^b^*	23.93 ± 0.36^b^*^c^*	21.61 ± 0.61^b^*^c#^	18.32 ± 0.49^b^*^c^*^d†^	25.92 ± 0.59^b^*
UAE (μg/24 hrs)	210.66 ± 0.32	1548.25 ± 0.90^a^*	491.23 ± 7.28^b^*	465.67 ± 4.02^b^*^c#^	452.09 ± 7.39^b^*^c^#d*	489.24 ± 9.04^b^*
Creatinine clearance	3.09 ± 0.07	3.75 ± 0.18	3.80 ± 0.18 ^b#^	5.23 ± 0.24 ^b^*	8.24 ± 0.58^b^*^c^*^d#^	8.93 ± 0.39^b^*
SOD (U/mg protein)	3.67 ± 0.08	1.23 ± 0.03 ^a^*	1.69 ± 0.03^b^*	2.19 ± 0.02^b^*^c^*	3.17 ± 0.06^b^*^c^*^d^*	3.41 ± 0.03^b^*
GSH (μM/mg protein)	69.04 ± 0.47	36.07 ± 0.44 ^a^*	40.01 ± 0.51^b^*	48.99 ± 0.35^b^*^c^*	56.37 ± 0.39^b^*^c^*^d^*	60.51 ± 0.49^b^*
TBARS (nmol/mg protein)	0.57 ± 0.02	2.29 ± 0.03^a^*	2.11 ± 0.01^b^*	1.91 ± 0.03^b^*^c†^	1.60 ± 0.02^b^*^c^*	1.69 ± 0.03^b^*
AGEs (RFU/mg protein)	1.62± 0.06	3.78± 0.09^a^*	3.24± 0.06^b^*	2.80± 0.05^b^*^c#^	2.64± 0.08^b^*^c^*	3.50± 0.09^b^*

Values are expressed as mean ± S.E.M. (n = 6 in each group).

### Improvement in liver enzymes

STZ-induction leads to fatty liver which is associated with hypercholesterolemia and hypertriglyceridemia. Fatty liver is characterised by elevated levels of liver enzymes i.e. Aspartate aminotransferase (AST) and Alanine aminotransferase (ALT). The elevated levels of AST and ALT were significantly reversed by CPE (100, 200 and 400 mg/kg) and glimepiride (10 mg/kg) as well**(Table 2)**. These resultsreveal the hepatoprotective effects of CPE.Studies conducted by Smojlik et al., 2010; Moustafa et al., 2012 andÖzbek et al., 2016 [28,29,30] have also evidenced the hepatoprotective effects of *C*. *sativum* and hencesupport our results.

### Improvement in kidney index and other renal parameters

Chronic hyperglycemia and oxidative stress leads to structural as well as functional abnormalities. Renal structural abnormalities (basement membrane thickening, mesangial expansion, and hypertrophy) leads to elevated kidney index (kidney weight to body weight ratio) in STZ-diabetic rats as compared to those in the normal control group. Oral administration of diabetic groups with 100, 200 and 400 mg/kg CPE has significantly attenuated kidney index (0.57 ± 0.006, 0.52 ± 0.007 and 0.49 ± 0.005 respectively) in comparison to DN control rats (0.98±0.016).

Functional abnormalities such as increased levels of albumin, urea, uric acid, creatinine and BUN represents onset of DN. Increased levels of albumin in urine (albuminuria) is related to abnormal lipid metabolism in DN[31]. Significant increase of urine albumin levels in diabetic rats, representing that albuminuria was related to deteriorating kidney function and altered lipid metabolism. Treatment with CPE normalized these levels therefore, exhibiting its beneficial role against albuminuria.

In the present study,biomarkers such as serum urea, uric acid, creatinine, BUN, and UAE were found to elevated which indicated the onset of DN. Oral administration of CPE has reversed the increased renal parameters and these attenuating effects were found to be dose dependent. Diabetic rats were also found with increased urine volume and urinary creatinine in comparison to normal control. These levels were significantly reduced by treatment with CPE (100, 200 and 400 mg/kg) along with an improvement in Ccr. **(Table 2).**

### Inhibition of AGEs

Chronichyperglycemiaresults to increased formation of AGEs and their receptors. AGEs together with ROS play major role in kidney damage. Various conducted studies confirm the inhibition of AGEs plays a vital role in attenuation of diabetic complications[31,32], therefore, AGEs inhibition could be considered as a good therapeutic approach that may alter pathogenesis and delay the progression of DN. CPE exhibits inhibitory effect against formation of AGEs in kidney as compared to DN control rats(**Table 2**).

### Effect of CPE on antioxidant enzymes and TBARS

Chronic hyperglycemia activates several biochemical pathways which collectively produces free radicals and ROS and ultimately to declined level of antioxidant enzymes and increased lipid peroxidation. STZ administration has also produced significant reduction in renal antioxidant enzymes (SOD and GSH) and increased lipid peroxidation in terms of TBARS levels. Petroselinic acid is the major fatty acid of coriander seeds ranging from 65 to 76%[3]. Petroselinic acid scavenges free radicals and therefore inhibits lipid peroxidation. CPE enriched with Linalool and ascorbic acid like potent antioxidants has significantly reduced lipid peroxidation and level of antioxidant enzymes was also found to be high as compared to diabetic control rats (**Table 2**).Additionally, linalool could be used in therapeutic approaches for the treatment of complications due to oxidative damage[33,34,35]. The antioxidant potential of coriander is also evidenced[4,28,35] which has supported our results in a positive manner.

### Histopathology

**Fig 3:**
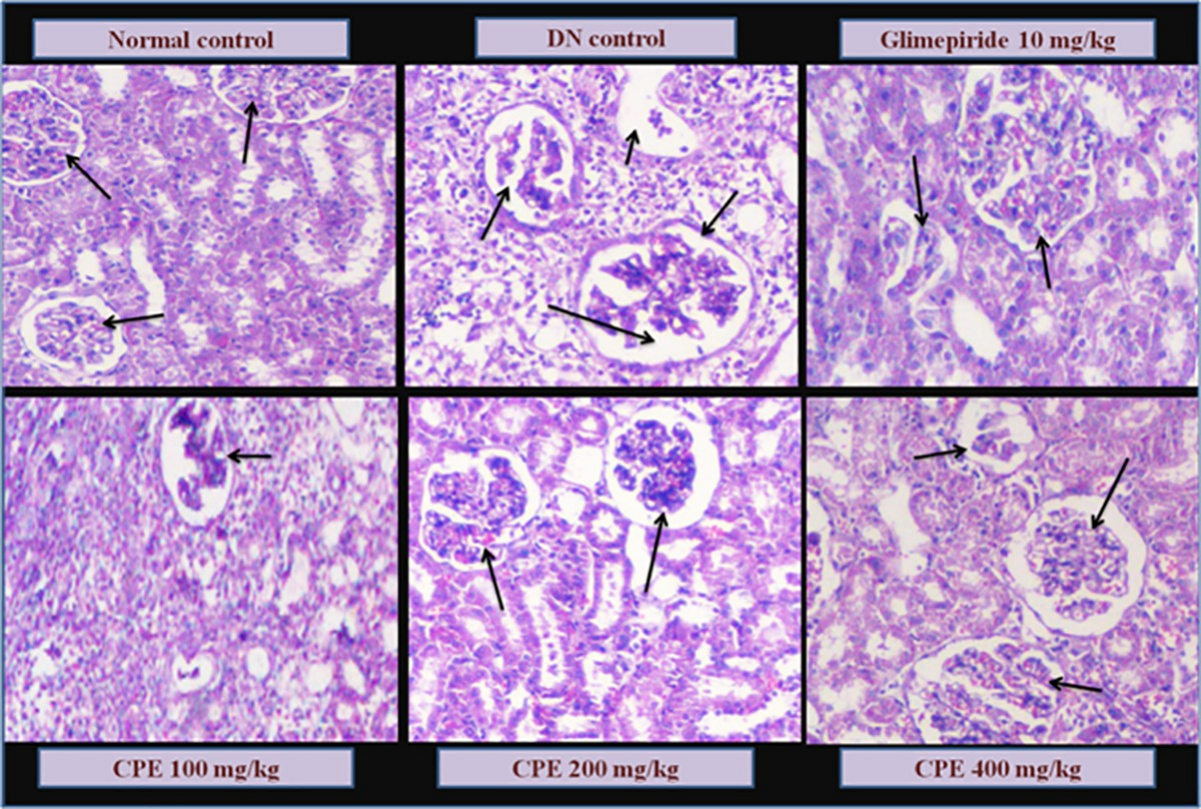
Histopathological changes in Kidney of normal, diabetic control, CPE and standard (Glimepiride) treated rats (H&E × 100). Arrows indicating the normal structure of glomerulus in Normal control; Mesangial cell expansion and hypertrpophy leading to destruction of normal glomerulus in DN rats; Regaining the structures near normal and atrophy with treatment of glimepiride and different doses of CPE (100, 200 and 400 mg/kg).

Kidneys were collected for histopathological examination. Compared with the normal control, DN control group showed signs of pathological markers such as mesangial expansion and thickening of glomerular capillaries along with increased glomerular space and atrophy of glomeruli. Normal control rats showed normal architecture, glomerular size and basement membrane thickness. Glimepiride treatment group has reduced necrotic condition in convoluted tubules with reduced infiltration of inflammation cells in cortex and medulla. However, morphology of the glomerulus was improved by CPE treated group *viz*., reduction in mesangial expansion, membrane thickness and atrophy with different doses. Linalool has been studied for its beneficial effects on renal tissues[34]which may have contributed to structural changes for CPE treated rats (**Fig 3**).

Thus, antioxidant compounds and fatty acids present in the plant might be helpful in the amelioration of chronic blood glucose level, oxidative stress *via* inhibition of AGEs and its related consequences and may be effective against progression of DN.

### Molecular docking studies

**Fig 4:**
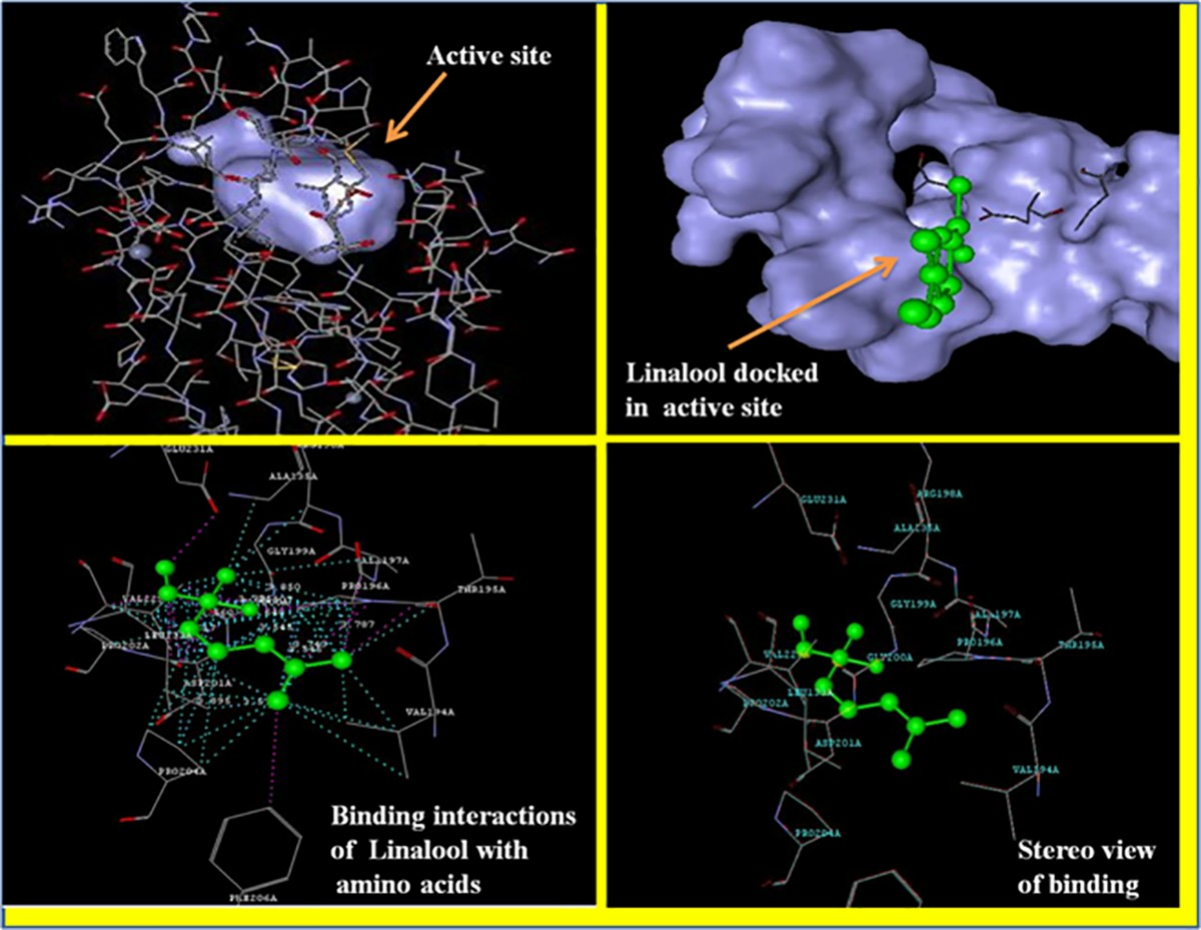
Binding modes of Linalool with RAGEs (PDB:3CJJ). Linalool has been shown to bind with active sites of RAGEs and interacting with the amino acids.

Strong evidences have emerged in recent years in support of an association between AGEs and diabetic complications. Excess of glucose undergoes mailard reactions for the generation of intermediate Schiff bases and amadori products like glucosepane, fructosamine etc. which are further converted to AGEs and consequently injures the organs and tissues. AGEs acts through their receptors known as RAGEs or receptor for AGEs. Selective prevention of AGEs by using agents which may bind to RAGEs, therefore blocks the damaging action of AGEs. This could be an interesting therapeutic approach for attenuation of DN [10]. In order to reveal the mechanism, we have docked the most active constituent of our extract *i*.*e*., Linalool to RAGEs (PDB ID: 3CJJ) crystal structure. Linalool has shown a good binding interaction with a dock score of -2.098 kcal/mol. Linalool was found to be interacted with amino acid residues of RAGEs including GLY_200_ and GLY_199,_ VAL_197_ and VAL_229_, ASP_201_, ALP_135_ with a distance of lower than 2.45 Å. The binding interactions are shown in **Fig 4**.

## Conclusion

Present study revealed that administration of petroleum ether extract of *C*. *sativum* seeds has ameliorated hyperglycemia, oxidative stress, hyperlipidemia as well as formation of AGEs which may be a possible contribution of its phytoconstituents like terpene and fatty acids. Thus CPE exhibits protective action in STZ-induced diabetic nephropathy. Moreover, further researchto elucidate detailed mechanism of *C*. *sativum* at the cellular and molecular levels in diabetic nephropathy needs to be done.

## Supporting information

S1 Fig Developed TLC plate of CPE under UV-light. (PDF)

S2 Fig TLC-bioautograph of CPE indicating the presence of antioxidant compounds. (PDF)

S3 Fig HPLC Chromatogram for Linalool (standard, A) and CPE (B) indicating the peak of linalool at near about similar retention time (RT).
(PDF)
