# Supplementary figures and images for: Coriandrum sativum seeds extract mitigate progression of diabetic nephropathy in experimental rats via AGEs inhibition
Source: PLoS One. 2019 Mar 7;14(3):e0213147. doi: 10.1371/journal.pone.0213147 (PMC6405108; doi:10.1371/journal.pone.0213147)

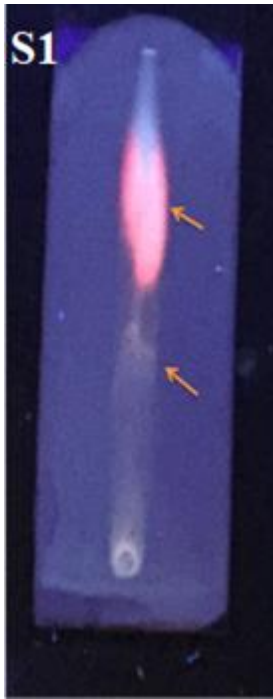

S1 Fig

Supplement: S1 Fig — (PDF) [file pone.0213147.s001.pdf]

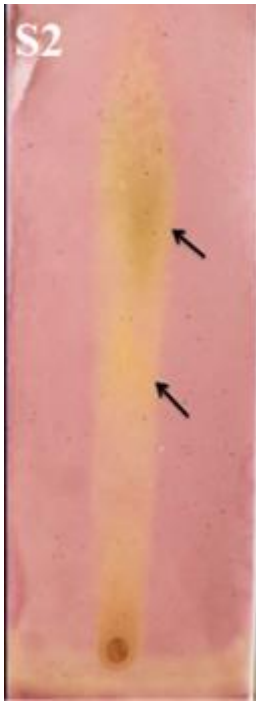

S2 Fig

Supplement: S2 Fig — (PDF) [file pone.0213147.s002.pdf]

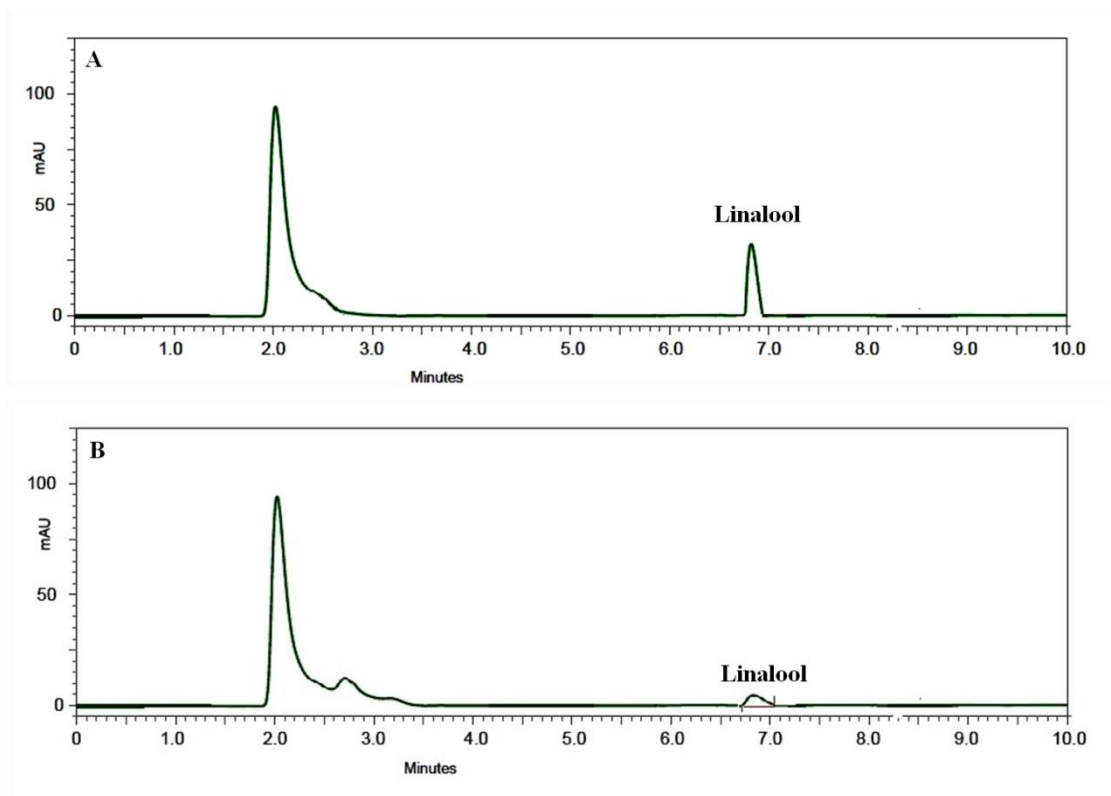

S3 Fig

Supplement: S3 Fig — HPLC Chromatogram for Linalool (standard, A) and CPE (B) indicating the peak of linalool at near about similar retention time (RT). (PDF) [file pone.0213147.s003.pdf]
